# Immune Reconstitution after Allogeneic Hematopoietic Stem Cell Transplantation

**DOI:** 10.3389/fimmu.2016.00507

**Published:** 2016-11-17

**Authors:** Justyna Ogonek, Mateja Kralj Juric, Sakhila Ghimire, Pavankumar Reddy Varanasi, Ernst Holler, Hildegard Greinix, Eva Weissinger

**Affiliations:** ^1^Transplantation Biology, Department of Hematology, Hemostasis, Oncology and Stem Cell Transplantation, Hannover Medical School, Hannover, Germany; ^2^BMT, Department of Internal Medicine I, Medical University of Vienna, Vienna, Austria; ^3^Department of Hematology and Oncology, University of Regensburg, Regensburg, Germany; ^4^Division of Hematology, Medical University of Graz, Graz, Austria

**Keywords:** hematopoietic stem cell transplantation, immune reconstitution, infection, graft-versus-leukemia effect, graft-versus-host disease

## Abstract

The timely reconstitution and regain of function of a donor-derived immune system is of utmost importance for the recovery and long-term survival of patients after allogeneic hematopoietic stem cell transplantation (HSCT). Of note, new developments such as umbilical cord blood or haploidentical grafts were associated with prolonged immunodeficiency due to delayed immune reconstitution, raising the need for better understanding and enhancing the process of immune reconstitution and finding strategies to further optimize these transplant procedures. Immune reconstitution post-HSCT occurs in several phases, innate immunity being the first to regain function. The slow T cell reconstitution is regarded as primarily responsible for deleterious infections with latent viruses or fungi, occurrence of graft-versus-host disease, and relapse. Here we aim to summarize the major steps of the adaptive immune reconstitution and will discuss the importance of immune balance in patients after HSCT.

## Introduction

**Figure 1:**
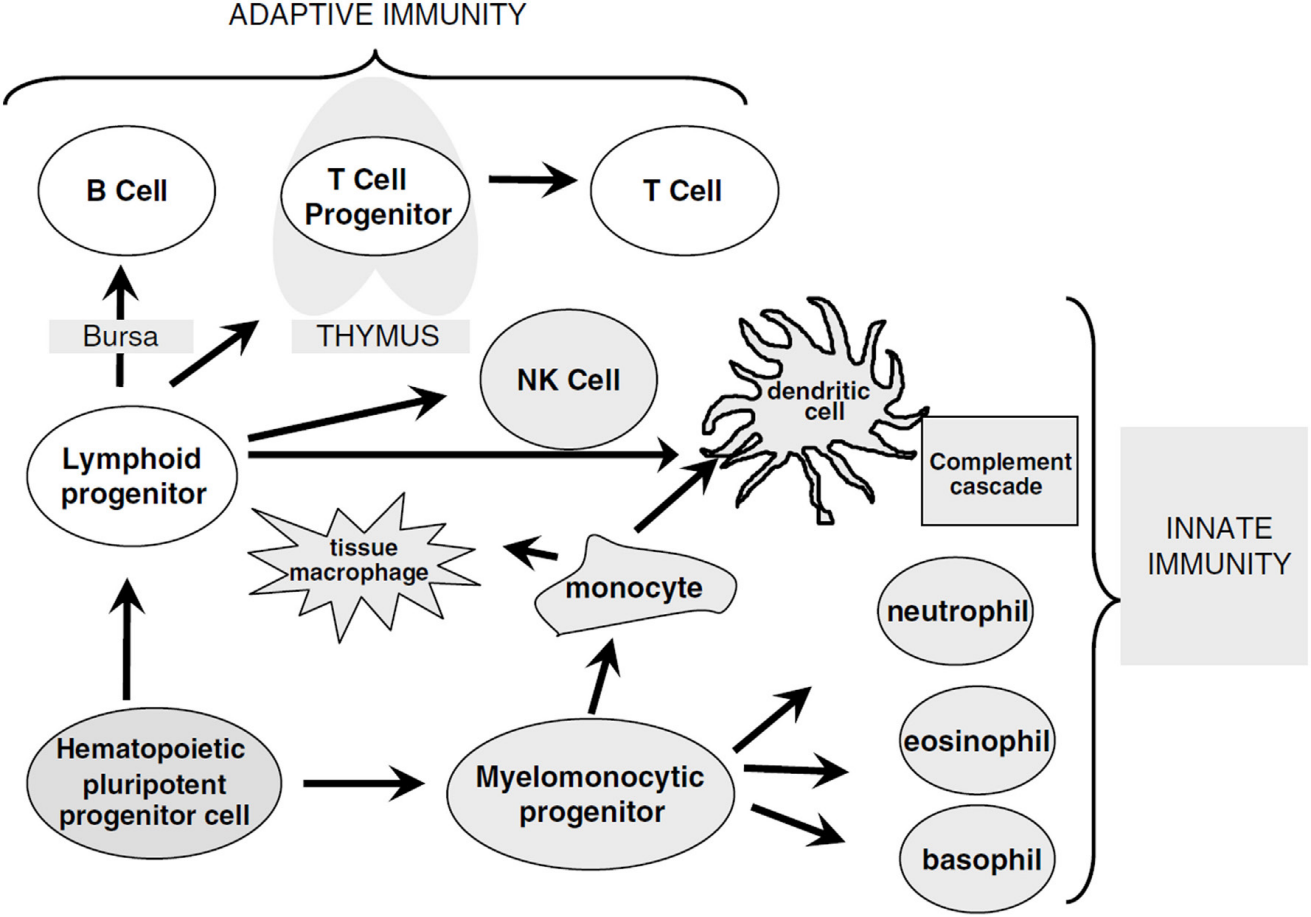
**Overview of immune cell differentiation**. The figure shows the different types of immune cells and their development from different precursors. The reconstitution of innate immunity occurs rapidly within 20–30 days after allogeneic HSCT while reconstitution of adaptive immunity is delayed following HSCT and can require up to 1 year. Natural killer (NK) cells, monocytes, granulocytes, and dendritic cells are derived from myelomonocytic progenitor cells. B and T cells differentiate from lymphoid progenitor cells and require specialized microenvironments in order to efficiently differentiate from primitive progenitors, and typically show delayed and incomplete recovery. Reprinted by permission from Macmillan Publishers Ltd.: Bone Marrow Transplantation (5), copyright (2005).

The reconstitution of different immune cell subsets after allogeneic hematopoietic stem cell transplantation (HSCT) (Figure 1) occurs at different time points summarized in Table 1. After conditioning therapy, patients undergo an “aplastic phase” (severe neutropenia or pre-engraftment phase) until neutrophils recover. The total nucleated cell (TNC) dose and CD34^+^ cell dose within the graft source are important factors contributing to the rate of engraftment and outcome after HSCT. Umbilical cord blood (UCB) grafts contain lower TNC levels compared to bone marrow transplant (BMT) and peripheral blood stem cell transplant (PBSCT), what increase the time of neutrophil engraftment from ~14 days after PBSCT and 21 days after BMT to 30 days after UCB transplantation (1, 2). Moreover, recent study showed that high TNC cell dose was associated with improved overall survival (OS), decreased relapse, and increased incidence of chronic graft-versus-host disease (GvHD) in patients receiving PBSCT (3). On the other hand, it has been presented that patients with higher CD34^+^ dose within PBSCT had faster platelet engraftment, but lower OS and increased relapse (4).

**Table 1:** **Immune reconstitution after allogeneic HSCT**.

Immune cells	Duration after allogeneic HSCT
Neutrophils >0.5 × 10^9^/L	~14 days for PBSC, ~21 days for BM, and ~30 days for CB
NK cells	30–100 days
T cells	100 days
CD19^+^ B cells	1–2 years

*PBSC, peripheral blood stems cells; BM, bone marrow; CB, cord blood; NK cells, natural killer cells*.

**Figure 2:**
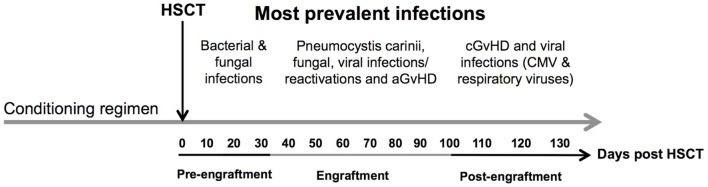
**Time line of complications after allogeneic HSCT**. The figure shows the most prevalent complications after HSCT according to the three phases of engraftment. Concomitant infectious complications consisting of bacterial, fungal, and viral infections are shown according to their occurrence as well as association with acute and chronic GvHD during different phases of follow-up: (1) pre-engraftment, (2) engraftment, and (3) post-engraftment phase. Abbreviations: CMV, cytomegalovirus; aGvHD, acute graft-versus-host disease; cGvHD, chronic graft-versus-host disease.

The infections encountered during the pre-engraftment phase consist primarily of bacterial and fungal infections that are reasonably well controlled by medications given for prophylaxis and treatment (6) (Figure 2). The first 100 days after HSCT (engraftment phase) are characterized by cellular immunodeficiencies due to a reduced number of natural killer (NK) cells of the innate immune system and T cells of the adaptive immune system. This renders patients especially susceptible to viral reactivations including cytomegalovirus (CMV) and Epstein–Barr virus (EBV) as well as viral diseases (7, 8).

The recovery of the T cell compartment relies on peripheral expansion of memory T cells, driven by cytokines as well as allogeneic antigens encountered in the host, and is followed by the production of naive T cells in the thymus (5). CD4^+^ T cells reconstitute later than CD8^+^ T cells and depend more on thymic generation of CD4^+^CD45RA^+^ naive T cells after HSCT explaining the reported inversion of the CD4/CD8 ratio (9). About 3 months after HSCT, CD4^+^ T cell numbers of about 200/μL have been observed (10). T cell receptor (TCR) rearrangement excision DNA circles (TRECs) have been investigated as surrogate parameters for reconstitution of thymus-derived CD4^+^CD45RA^+^ naive T cells (11). TREC levels remain low until 3–6 months after allogeneic HSCT (5). A special subgroup of CD4^+^ cells are regulatory T cells (Tregs), which may be important for a better outcome after allogenic HSCT (12). Tregs suppress the activity of effector T cells, thus reducing inflammation and promoting immune homeostasis after allogenic HSCT (13). Clinical, preclinical, and experimental models have shown that Treg reconstitution plays a critical role in amelioration of GvHD while preserving the graft-versus-leukemia (GvL) effect (14, 15). Increasing age is associated with thymic atrophy and loss of function (16). Cycling of mature lymphocytes maintains numbers of mature T cells by homeostatic peripheral expansion (5). Naive CD4^+^ and CD8^+^ T cells rely on interleukin-7 (IL-7) and TCR engagement for survival and expansion (17). CD8^+^CD27^+^ memory T cells can be maintained and expanded by cytokine signals alone involving IL-7 and interleukin-15 (IL-15) (18). In older patients, the lack of CD4^+^CD45RA^+^ naive T cells with a broad TCR repertoire leads to an increased risk for opportunistic infections and probably also to increased risk of leukemic relapse (19, 20). The lack of CD4^+^CD45RA^+^ naive T cells is additionally aggravated by GvHD (21, 22).

The B cell compartment representing the humoral immunity is the slowest to reconstitute and may take up to 5 years after allogeneic HSCT. Transitional CD19^+^CD21^low^CD38^high^ B cells are the first B cells emigrating from the bone marrow (BM) and are elevated in the peripheral blood (PB) in the first months after HSCT before their percentage progressively decreases, while the proportion of more mature B cell subpopulations increases (23). The lack of CD19^+^CD27^+^ memory B cells, decreased levels of circulating immunoglobulins, impaired immunoglobulin class switching, and a loss of complexity in immunoglobulin gene rearrangement patterns leave allogeneic HSCT patients vulnerable to encapsulated bacteria such as *Streptococcus pneumoniae* and *Haemophilus influenzae* (1, 24). In this review, we summarize the reconstitution of the adaptive immunity and discuss the importance of achieving immune balance after HSCT.

## Adaptive Immunity

### Immune Reconstitution of B Cells after Allogeneic Hematopoietic Stem Cell Transplantation

Patients undergoing HSCT often experience late recovery of B cell numbers leading to a defect of B cell mediated immunity. Generally, B cell numbers recover to normal counts within 12 months after HSCT (25), although complete recovery may take up to 2 years. In the first few months, very few circulating B cells have been observed (25, 26) and within 1–2 years following HSCT, B cell numbers reach levels exceeding normal adult individual ones followed by gradual decline, similarly to the normal ontogeny in young children (26). First B cells emerging into the periphery are CD19^+^CD21^low^CD38^high^ transitional B cells, which subsequently decrease in percentages while mature CD19^+^CD21^high^CD27^−^ naive B cells are being replenished (1, 23). Transitional B cells were first described as CD24^high^CD38^high^ (23). Later on, another marker of transitional B cells was identified, distinguishing between T1 and T2 transitional cells. T1 cells were reported as CD21^low^ and described as the first B cell population emigrating from the BM, which subsequently differentiate toward CD21^+^, T2 phenotype and serve as precursors of the CD19^+^CD21^high^CD27^−^ naive B cell pool in PB and tissues (27). Complete reconstitution of the B cell compartment includes the recovery of both CD19^+^CD21^high^CD27^−^ naive and CD19^+^CD27^+^ memory B cells. Reconstitution of memory B cells occurs upon environmental or vaccine-based antigen exposure and requires CD4^+^ T cell help (28). Complete CD19^+^CD27^+^ memory B cell development may take up to 5 years after HSCT (26). In the study by Corre and colleagues, numbers of CD19^+^CD21^high^CD27^−^ naive B cells normalized by 6 months and reached above normal values around 24 months after myeloablative conditioning for allogeneic HSCT (29). CD19^+^CD27^+^ memory B cells remained persistently low during the 2 years of follow-up (29). Other authors similarly reported relatively fast naive B cell reconstitution followed by delayed memory B cell recovery (30, 31). In addition, early expansion of CD19^+^CD5^+^ B cells has been reported (29, 32), a subset described as pre-naive circulating B cells representing a distinct intermediate phenotype between transitional and naive B cells (33). These cells showed only partial responses to B cell receptor (BCR) stimulation and CD40 ligation, but similarly to CD19^+^CD21^high^CD27^−^ naive B cells, these were capable to differentiate into plasma cells and had the ability to function as antigen-presenting cells (APCs) (33).

In the first 2 years following allogeneic HSCT, B cell function remains compromised. Different B cell subpopulations often reconstitute over a different period of time contributing to a defective humoral response. Delayed T cell recovery and the reversed CD4/CD8 ratio may also contribute to low circulating B cell numbers following HSCT (26). Furthermore, CD19^+^CD27^+^ memory B cells can be influenced by low T helper cells as they require their help for isotype switching (26). In addition, somatic hypermutation seems to be diminished even in the presence of normal donor CD4^+^ T cell numbers, implying an environmental defect (26, 34). Normal levels of serum IgM are usually measurable 3–6 months after HSCT (35, 36), followed by normalization of serum IgG1/IgG3, IgG2/IgG4, and IgA similar to that observed during normal development in the first years of life (37). However, in some patients, long-term antibody class deficiencies have been reported (38). The immunoglobuline heavy chain (IgH) repertoire is often characterized by delayed class switching and oligoclonal dominance with specific rearrangements dominating at different time points in these patients (36, 39). Measurement of B lymphocyte repertoire diversity by analysis of IgH complementarity determining region 3 (CDR3) revealed limited variation of IgH CDR3 repertoire in CD19^+^CD27^+^ memory B lymphocytes compared to CD19^+^CD21^high^CD27^−^ naive B cells at 3 and 6 months after allogeneic HSCT. Decrease in CD19^+^CD27^+^ memory B cell IgH CDR3 repertoire, but not CD19^+^CD21^high^CD27^−^ naive B cell one, was also observed when compared to healthy controls suggesting a role of CD19^+^CD27^+^ memory B cells in oligoclonal restriction (35). Both CD19^+^CD27^+^ memory B cells and CD19^+^CD21^high^CD27^−^ naive B cells reach normal diversity, comparable to healthy individuals, 12 months after HSCT (35).

Different settings of HSCT may also influence B cell recovery. Patients receiving antithymocyte globulin-fresenius (ATG-F) presented delayed CD19^+^ B cell recovery up to 5 months after HSCT compared to non-ATG-F patients (40). ATG is a potent immunosuppressant administrated before HSCT to prevent graft rejection and to reduce incidence of acute and chronic GvHD in patients receiving grafts from unrelated donors (40, 41). Absolute CD19^+^ B cells normalized 1 year after HSCT in both groups. ATG-treated patients had significantly worse CD19^+^CD21^high^CD27^−^ naive B cell and CD19^+^CD27^+^ memory B cell regeneration within the first month after HSCT indicating a negative impact of ATG on B cell immune reconstitution (40). Depending on the brand, ATG may also have immunomodulatory effects on B cells (42). Slow B cell recovery has been observed in patients receiving non-myeloablative conditioning compared to those given myeloablative therapy, with reduced B cell numbers observed in most patients up to 12 months after non-myeloablative therapy for HSCT (43). However, these findings may in part be explained by older patient age and higher incidence of acute GvHD in this patient cohort (43). Both acute and chronic GvHD have been associated with delayed B cell reconstitution, and reduction or lack of B cell precursors in the BM has been observed in these patients compared to patients without GvHD (44). In a study on 93 allograft recipients, the number of BM B cell precursors on day 30 after HSCT was significantly lower in patients later developing grades 2–4 acute GvHD compared to patients with grades 0–1 disease (44). Moreover, patients developing extensive chronic GvHD within 1 year after transplantation had lower percentages of B cell precursors on day 365 compared with patients without chronic GvHD or with limited chronic GvHD (44). However, the effect of acute and chronic GvHD could not be separated from the possible influence of glucocorticoid treatment in this study due to low patient numbers suggesting B cell deficiency after transplantation may in part be a result of inhibition of B lymphopoiesis by GvHD and/or its treatment (44). In addition, a decrease of absolute CD19^+^ B cells in patients at first diagnosis of chronic GvHD and a disturbance of B cell homeostasis in patients with active chronic GvHD have been observed (45, 46). Stem cell source may also influence numbers of circulating B cells with higher counts detected in recipients of peripheral blood stem cells (PBSC); however, this observation may be attributed to the higher amount of mature B cells in PBSC grafts compared with BM (44, 47, 48).

Even patients who show recovery of overall CD19^+^ B cell numbers are not considered fully immunocompetent and as a result of decreased B cell function, impaired vaccine responses to infectious antigens have been observed (26). Lack of CD19^+^CD27^+^ memory B cells, decrease of circulating immunoglobulins, and impaired immunoglobulin gene rearrangement render these patients susceptible to encapsulated bacteria and viruses (1, 24).

### Immune Reconstitution of T Cells and Their Role after HSCT

CD4^+^ and CD8^+^ T cells reconstitute within the first year after HSCT and enable defense against viral or fungal infections, as well as maintaining the GvL effect. A subset of CD4^+^ T cells are so called regulatory T cells (Tregs). In the next paragraph, we aim to summarize their development and function in patients after allogeneic HSCT.

### Regulatory T Cells in Immune Reconstitution and Their Impact after HSCT

#### Regulatory T Cells

**Figure 3:**
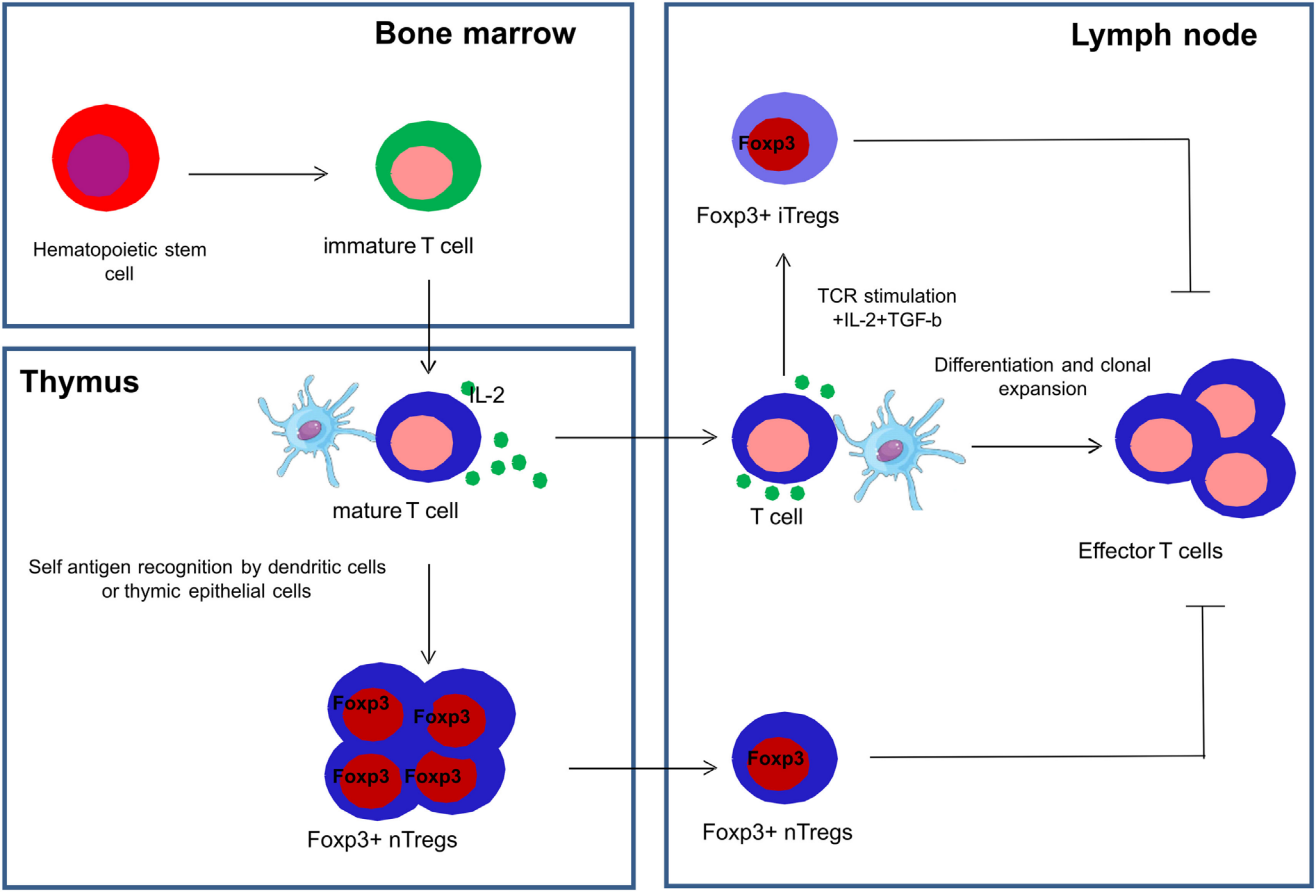
**Development of natural and induced regulatory T cells**. Natural regulatory T cells (nTregs) are derived from the thymus and are characterized by the co-expression of CD4, high expression of CD25 and FoxP3, and are collectively represented as CD4^+^CD25^+^FoxP3^+^ Tregs. Induced or adaptive regulatory T cells (iTregs) are generated in the peripheral lymphoid organs in the presence of transforming growth factor beta (TGF-β) and interleukin-2 (IL-2).

Tregs are a subset of CD4^+^ T cells whose function is to suppress immune responses and maintain self-tolerance (49). A transcription factor called FoxP3, a member of the fork head family of transcription factors, is critical for the development and function of Tregs and is used as a definite marker to identify Tregs (49, 50). Tregs are a functionally mature subpopulation of T cells and can also be induced from CD4^+^CD45RA^+^ naive T cells in the periphery (51). Natural Tregs (nTregs) are derived from the thymus and are characterized by the co-expression of CD4, high expression of CD25 and FoxP3 (52). Induced or adaptive Tregs (iTregs) are generated in peripheral lymphoid organs in the presence of transforming growth factor beta (TGF-β) (53) (Figure 3).

Some recent studies have shown that nTregs are more stable than iTregs in relation to their differential DNA methylation profiles and other epigenetic regulations of FoxP3 (54, 55).

#### Tregs in Immune Balance

Tregs can downregulate immune responses by (a) production of inhibitory cytokines and (b) a contact-mediated effect on APCs. Tregs produce the anti-inflammatory cytokine interleukin-10 (IL-10) that inhibits production of interleukin-12 (IL-12) by activated dendritic cells (DCs) and macrophages (56, 57). IL-10 also inhibits the expression of co-stimulators and major histocompatibility complex (MHC) class II molecules on DCs and macrophages and thus inducing tolerance within the immune system (56–58). Another anti-inflammatory cytokine produced by Tregs, TGF-β, inhibits the proliferation and effector functions of T cells and the activation of macrophages (59, 60). TGF-β also regulates the differentiation of functionally distinct subsets of T cells, stimulates production of immunoglobulin A (IgA) antibodies, promotes tissue repair after local immune and inflammatory reactions subside, and confers Treg-mediated immune reconstitution (56–58, 61). Tregs play a major role in regulation of epithelial inflammation and are strongly influenced by the interaction with the epithelial microbial environment (62, 63).

### Tregs in Animal Models of Hematopoietic Stem Cell and Solid Organ Transplantation

Tregs play an indispensable role in both solid organ transplant tolerance and in allograft tolerance after HSCT. In rodents and humans, a subpopulation of thymus-derived naive CD4^+^ T cells that co-express the IL-2R alpha chain, CD25, have potent suppressive activity (64). Tregs mediate transplantation tolerance in experimental models of skin and/or solid organ transplantation (65) as well as tolerance to BM allografts (66). By allogeneic HSCT, malignant and non-malignant hematological disorders can be cured, but at the same time, treatment efficacy is limited due to occurrence of GvHD (67). Regulatory T cells have received considerable attention in recent years due to their ability to suppress the proliferation of conventional T cells when added to donor grafts and prevention of GvHD in animal models (68). Using a mouse model, Edinger and colleagues have shown that CD4^+^CD25^+^ Tregs suppress GvHD after BMT without abrogating the GvL or graft-versus-tumor (GvT) effect (14) supporting the importance of Tregs in allogenic HSCT. Furthermore, Nguyen and colleagues demonstrated that the adoptive transfer of Tregs preserved thymic and lymphoid architecture of the host and hence accelerated posttransplant T cell immune reconstitution in a murine GvHD model (69).

Taylor and colleagues demonstrated that *in vivo* depletion of CD25^+^ T cells and depletion of CD25^+^ T cells in the transplant inoculum, worsened GvHD whereas adoptive transfer of CD4^+^ CD25^+^ nTregs together with the BM graft ameliorated GvHD (70).

While an increasing number of publications have focused on the biology of CD4^+^CD25^+^Foxp3^+^CD45RO^lo^ naive Tregs in GvHD, less attention has been given to iTregs, probably due to the lack of proven cell surface marker that differentiate nTregs from iTregs. Fantini and colleagues demonstrated that iTregs can be generated from CD4^+^ T cells in the presence of TGF-β and can be expanded in culture (71). On the other hand, Koenecke and colleagues showed that administration of *in vitro* generated iTregs along with BM grafts containing alloreactive donor T cells did not provide any significant protection from lethal GvHD, due to limited *in vivo* survival of these cells (72). They also demonstrated that iTregs lost their Foxp3 expression, along with a loss of suppressive function early after transplantation, thus making iTregs unsuitable for use in a therapeutic approach (72) if administered as an external cellular product. Not only iTregs but also nTregs have been shown to loose Foxp3 expression in a STAT3-dependent manner and can revert to a proinflammatory phenotype under inflammatory conditions (73). Therefore, inflammation seems to affect Foxp3 expression in both natural and induced Tregs.

Although Tregs have long been assumed to be solely a subset of the CD4^+^ T cell compartment, a CD8^+^ Treg population has been recently described and shown to be capable of suppressing T cell responses (74). In terms of GvHD, Robb and colleagues reported that CD8^+^Foxp3^+^ Tregs suppressed GvHD and attenuated GvHD mortality after BMT in a mouse model (75). Interestingly, CD8^+^Foxp3^+^ cells were more suppressive than CD4^+^Foxp3^+^ cells (75). Using a rat model, Xystrakis and colleagues provided a first report on CD8^+^ Tregs conferring their regulatory properties *via* a cell to cell contact dependent mechanism to prevent GvHD and thus confirming CD8^+^Foxp3^+^ Tregs in a second species (76). Clinical studies on CD8^+^ Tregs at a functional level are scarce to date. However, Zheng and colleagues reported that human CD8^+^ Tregs potentially inhibit GvHD without compromising general immunity and GvL activity in humanized mouse models (77). Taken together, these findings provide an insight into the efficacy of both CD4^+^ and CD8^+^ Tregs as potential novel therapeutic approaches in clinic.

### Tregs in Clinical Hematopoietic Stem Cell Transplantation

Many researchers have focused on evaluating Treg cell numbers after HSCT, since they play an important role in the amelioration of GvHD. Using PB of patients after transplantation, Li and colleagues demonstrated that the frequency of CD4^+^CD25^+^ Tregs was significantly downregulated in patients with severe acute or chronic GvHD (78). They also showed that a decreased level of CD4^+^CD25^+^ Tregs correlated with increased severity of GvHD (78). While the majority of studies focused on blood derived Tregs, there is little information on Tregs isolated from intestinal tissues due to the lack of availability of repeated gut biopsies. Using immunoenzymatic labeling, Rieger and colleagues were the first to demonstrate that infiltrating Tregs decreased the signs of acute and chronic GvHD in intestinal mucosa (79). They showed that patients with acute and chronic GvHD had a complete lack of counter regulation indicated by a Foxp3^+^/CD8^+^ T cell ratio identical to that of healthy individuals, while this ratio was increased in patients without GvHD (79). These results have been discussed controversially in the literature since Lord and colleagues demonstrated that Foxp3^+^ T cells were not decreased in PB or gastrointestinal tissues and that the frequency of Tregs did not correspond to disease incidence or severity (80). On the contrary, these investigators reported that Foxp3^+^ T cells were significantly upregulated in GvHD-afflicted intestinal mucosa when compared to non-GvHD mucosal tissues (80). This finding was further supported by Ratajczak and colleagues who observed an increased proportion of CD4^+^Foxp3^+^ T cells in patients with grades 2–4 compared to grades 0–1 acute GvHD (81). One possible explanation for these conflicting results may be the difficulty to discriminate natural and induced Tregs. It is possible that nTregs are decreased in GvHD while iTregs may be increased in order to compensate for the exaggerated inflammation during GvHD. Imanguli and colleagues observed an upregulation of functional markers such as CD3^+^, CD4^+^, CD27^+^, ICOS^+^, and CD39^+^ in Tregs that traffic into tissue including skin and oral mucosa exerting a suppressive function in patients with chronic GvHD (82). Interestingly, normal numbers of activated CD45^−^Foxp3^hi^ Tregs were observed in tissue and PB of patients with chronic GvHD whereas naive or resting CD45RA^+^Foxp3^+^ Tregs that presumably control chronic GvHD effector cells were reduced compared to patients without chronic GvHD.

### Tregs in GvHD: First-in-Man Clinical Trial

Studies in mouse models of GvHD have provided information on the suppressive nature of Tregs and their potential to suppress and ameliorate GvHD without impairing the GvL effect. The first clinical trial using Tregs to suppress acute/chronic GvHD in patients were completed recently. This “first-in-man-study” reported the adoptive transfer of *ex vivo* expanded CD4^+^CD25^+^CD127^−^ Tregs in one patient with chronic GvHD and another with acute GvHD after HSCT with an HLA-identical sibling donor (83). Transfer of Tregs resulted in a reduction of the steroid dose administered, increased levels of circulating Tregs, and a decrease in inflammatory cytokine levels in the PB (83). Another “first-in-man-study” was reported after double UCBT in 23 patients, who received *in vitro* expanded 0.1–30 × 10^5^ UCB CD4^+^CD25^+^CD127^−^ Tregs per kilogram derived from partially HLA-matched third-party UCB units (15). There was a significant reduction in the incidence of acute GvHD grades II–IV (43 versus 61%, *P* = 0.05) when compared to 108 historical controls without transfusion of Tregs. No toxicities, infections, relapse, or early mortality were observed suggesting that UCB Tregs could be beneficial for preventing acute GvHD (15). Furthermore, Di Ianni and colleagues reported a clinical trial in 28 patients receiving adoptively transferred CD4^+^CD25^+^CD127^−^ Tregs after T-cell-depleted haploidentical HSCT without further immunosuppression (13). Only 2 out of 28 patients developed grades II–IV acute GvHD and no chronic GvHD was observed. They showed that adoptive transfer of freshly isolated donor-derived Tregs 4 days before inoculating the CD34^+^ stem cells prevented acute and chronic GvHD in the absence of further immunosuppression. Tregs promoted lymphoid reconstitution, improvement of immunity to opportunistic pathogens (no CMV-related death of patients) without abrogating the GvL effect (13). In addition, Hoffmann reported *in vitro* expansion of highly purified polyclonal human CD4^+^CD25^high^ Tregs through the use of artificial APCs for repeated stimulation *via* CD3 and CD28 in the presence of high-dose IL-2 (84). These cells not only maintained their phenotype and expressed suppressive activity but also maintained the expression of the lymph node homing receptors l-selectin and CCR7 (84). Furthermore, the same group reported results of a small phase I safety and feasibility trial where freshly isolated donor-derived CD4^+^CD25^high^ Tregs were infused into nine patients with high risk for leukemic relapse after cessation of systemic GvHD prophylaxis (12). After 8 weeks, additional CD4^+^ T cells were administered to promote GvL activity. Patients showed no signs of GvHD nor opportunistic infections or early disease relapse supporting the safety and efficacy of Treg transfusion (12). This has led to a phase II clinical trial for the treatment of patients with steroid-refractory acute GvHD using freshly isolated CD4^+^CD25^high^ Tregs that is currently ongoing. Taken together, these early trials suggest that Tregs could be a novel approach for prophylaxis and treatment of patients with acute GvHD in larger clinical trials. The impact of Treg transfusion on the immune reconstitution has to be further investigated.

### Induction of Regulatory T Cells after HSCT

Tregs induce tolerance and maintain immune homeostasis (51). A major challenge of Treg cell therapy is their relative scarcity in PB (0.5–1% of CD4^+^CD25^high^ T cells) (85). In 2011, Hippen and colleagues presented two individual reports regarding the generation of induced Tregs on a large scale (86) and *ex vivo* expansion of natural Tregs (86). Both methods focus on the development of expansion protocols for either type of Tregs to obtain higher yields for clinical trials on treatment or prevention of GvHD (86). In patients with chronic GvHD, Matsuoka and co-workers reported that daily administration of low-dose IL-2 induced selective expansion of functional CD4^+^CD25^+^CD127^−^ Tregs, improved chronic GvHD, restored CD4^+^ T cell homeostasis, and promoted the reestablishment of immune tolerance (87). Koreth et al. reported the case of 29 chronic GvHD patients that the administration of subcutaneous low dose IL-2 rapidly induced preferential and sustained Tregs expansion without any immune impairment (88). This suggests that low-dose IL-2 could be a potential therapy to restore immune balance after HSCT. Another approach to manipulate Tregs *in vivo* was reported by Furusawa and colleagues (89). Clostridial products, like short chain fatty acids (SCFA) or mainly butyrate, can induce the differentiation of colonic Tregs *in vitro* and *in vivo* in mouse models (89). This points toward the necessity of host–microbiome interaction to establish immunological tolerance and homeostasis in the gut. Moreover, Mathewson and colleagues reported that restoring clostridial metabolites or the strain itself modulated intestinal epithelial cell integrity and mitigated GvHD in mice (63). Taken together, these findings strongly suggest that the right balance of gut microbiome may be crucial to induce Tregs for intestinal tolerance.

### CD4^+^ and CD8^+^ T Cell Reconstitution

Memory T cells [central memory (T_CM_) and effector memory (T_EM_)], tissue resident memory cells (T_RM_), and effector cells (T_EFF_) cells are essential to control viral reactivations after allogeneic HSCT. Upon encountering antigens, memory cells differentiate to T_EFF_ and lyse the infected cells and secret proinflammatory cytokines [e.g., IFN-γ and tumor necrosis factor-α (TNF-α)] (90). Immune surveillance of T_CM_ occurs trafficking through secondary lymphoid organs, T_EM_ and T_EFF_, through non-lymphoid organs (91). In contrary, T_RM_ cells reside at various sites (e.g., liver, lungs, gut, and skin) and provide immediate antiviral response (cytotoxicity and secretion of IFN-γ) without trafficking (92). The reconstitution of CD4^+^CD45RA^+^ naive T cells, providing the broad range of TCR repertoire needed to control infections and to avoid the reappearance of leukemic cells, is essential after allogeneic HSCT (11, 93). The conditioning regimens applied, increasing patient age and occurrence of acute and chronic GvHD, have devastating effects on thymic function after HSCT (28, 93–95). Reconstitution of CD4^+^CD45RA^+^ naive T cells can be demonstrated by measuring TRECs. Immune reconstitution of CD4^+^ and CD8^+^ T cells is also essential for maintaining a GvL effect (1). Reconstitution of CD8^+^ T cells is faster than that of CD4^+^ T cells, which usually occurs around day +100 or later and is indicated by the inversion of the CD4^+^/CD8^+^ T cell ratio (1) early after HSCT (Table 1). The time period until complete reconstitution of CD4^+^ T cells can take up to 2 years after allogenic HSCT (96).

#### Major Factors Influencing T Cell Immune Reconstitution: GvHD and Immunosuppressive Treatment

Acute GvHD is one of the severe complications occurring early after HSCT contributing substantially to non-relapse mortality (NRM). Development of acute GvHD is influenced by human leukocyte antigen (HLA) disparities or gender mismatches between donor and recipient, the intensity of the conditioning regimen applied, CMV reactivation, and the stem cell source (97, 98). Acute GvHD can also occur in the HLA-identical transplant setting (siblings or matched unrelated donors) due to minor histocompatibility antigen differences between donor and recipient (98). Acute GvHD is an immune response directed against the host immune system, tissues, and organs (99, 100). GvHD by itself can inhibit T cell functions by limiting TCR diversity, T cell development, and dysfunction in cytokine production, most likely through damage of the BM and/or thymus, apoptosis, and release of cytokines in a so-called “cytokine storm” (101).

Bone marrow gives rise to all hematopoietic lineages and is the homing site for memory cells of the adaptive immunity (102). Recently, BM has been established as an additional target of alloreactivity observed during GvHD leading to the depletion of both hematopoietic progenitors and niche-forming cells (103), resulting in disrupted hematopoiesis and delayed immune reconstitution (104). Along with the BM, the thymus plays an important role in the maturation of hematopoietic precursors and T cell development (93). Acute GvHD substantially decreases thymic output and thus recovery of CD4^+^ T cells and diversified T cell repertoires (93). Acute GvHD leads to a further skewing of the TCR repertoires of both CD4^+^ and CD8^+^ T cells as well as antigen-specific T cells (99). Both T lymphopenia and inadequate repertoire of CD4^+^ and CD8^+^ T cells for at least 1 year after transplant foster recurrent infections with latent viruses.

In addition, treatment of patients with acute GvHD with corticosteroids or other immunosuppressive drugs increases the risk of viral reactivations (98, 105). It has been reported that the risk of CMV infections is directly related to the dose and duration of steroid administration (106). Administration of high doses of steroids was shown to be an independent risk factor for impaired functional recovery of CMV-specific CD4^+^ and CD8^+^ T cells (106). Moreover, Özdemir and colleagues reported that steroids induced a significant impairment of CD8^+^ T cells for producing TNF-α (107).

#### T Cell Depletion of the Stem Cell Graft

Although T cell depletion (TCD) of the stem cell graft reduces GvHD, it is associated with delayed immune reconstitution, infectious complications, and an increased risk of relapse (108). Thus, *ex vivo* T-cell depletion by either CD34^+^ cell selection or CD3^+^/CD19^+^ cell depletion has not been routinely performed and repletion protocols have been widely studied [e.g., HSV-Tk-transduced T cell transfer, other donor lymphocyte infusion-based protocols (109, 110)]. *In vivo* T cell reducing or impairing agents include ATG [e.g., ATG-Fresenius; Germany, or thymoglobulin (thymo); Genzyme; USA] or anti-CD52 antibody (alemtuzumab or campath), a particularly powerful reagent for immunosuppression (108, 111). ATG administration leads to prolonged immunosuppression of both CD4^+^ T cells and CD4^+^CD25^+^CD127^−^ Treg cells (111) and appears to have less severe effect on immune reconstitution when compared to campath (112).

#### Stem Cell Source

The source of stem cells can impact on both complications as well as time to immune reconstitution after allogeneic HSCT (Table 2). Investigators reported that the source of stem cells is a predictive factor for recovery of CMV-specific cytotoxic T lymphocytes (CTL) (10). Recipients of PBSCs had improved functional CMV–CTL recovery and earlier CMV-specific CD4^+^ T cell reconstitution than patients given BM grafts (106, 113). These findings can be explained by the fact that PBSC grafts compared to BM contain more lymphocytes and higher numbers of CD4^+^CD45RO^+^ memory T cells (114).

**Table 2:** **Stem cell source influences immune reconstitution and complications after HSCT**.

Complication	PBSCs	BM	CB
aGvHD	++	+	+/−
Infections	+	+	++
Viral reactivations	++	++	+/−
Relapse	+/−	++	++

*The table summarizes the influence of different stem cell sources on the immune reconstitution and selected complications after HSCT. The degree of association is indicated by plus (+)*.

*aGvHD, acute graft-versus-host disease; PBSCs, peripheral blood stem cells; BM, bone marrow; CB, cord blood; ++, high; +, moderate; and +/−, low*.

### Influence of CD4^+^ and CD8^+^ T Cells on GvL

Graft-versus-leukemia is defined as an immune response directed against leukemia/tumor cells of the recipient after allogeneic HSCT. Over the years, several studies have shown that CD4^+^ and CD8^+^ T cells play an important role in establishing a GvL effect through various mechanisms such as cytotoxic T cells releasing granzyme B and apoptosis mediated by FAS ligands (115). GvL is often associated with GvHD, but GvL responses against, e.g., minor histocompatibility antigens solely expressed on hematopoietic cells (mHA1) may be specifically directed against leukemic cells or the recipients’ hematopoietic cells. The precise role of CD4^+^ and CD8^+^ T cells for achievement of a GvL effect is not clearly understood today (115, 116). Complete depletion of T cells by CD34^+^ cell selection leads to a high incidence of relapse, resulting in death in about 20–50% of patients (117). T cell repletion or donor lymphocyte infusions (DLI) can prevent relapse, but can lead to a higher probability of acute and chronic GvHD (118, 119). Several protocols tried to circumvent the problem of increased acute and chronic GvHD by delayed add-back of genetically modified T cells (109) or other manipulations of the donor’s lymphocytes such as selection of either CD4^+^ or CD8^+^ T cells prior to transfusion (120, 121).

### Virus-Specific Immune Reconstitution (Antigen-Specific Cytotoxic T Cells) after HSCT

T cells are the most important effector cells in the control of viral infections. Thus, T cell reconstitution after allogeneic HSCT has a significant impact on the control of infectious complications. The first phase of virus-specific T cell reconstitution and expansion early after HSCT depends on the transfer of mature (effector, memory, or naive) virus-specific T cells within the donor graft and the resident antigen-specific cells (10, 122). Viral infections occur mostly between engraftment and day +90 posttransplant (123). However, also late (after day +90) and recurrent CMV reactivations have been observed, which have been associated with impaired reconstitution or function of antiviral immunity (106). CMV is a latent virus, which belongs to the family of herpesviruses and is one among the common viral pathogens that can reactivate after HSCT. It reactivates in about 60–70% of CMV-seropositive patients, and the primary infection affects 20–30% of CMV seronegative recipients transplanted from CMV-seropositive donors (124). Uncontrolled CMV reactivations can lead to a life-threatening, multi-organ CMV disease such as retinitis, gastroenteritis, or pneumonia (125–127). Advances in CMV monitoring, preemptive antiviral therapy, and quantification of CMV–CTLs are crucial in the prevention of CMV disease (128). The most important risk factors for CMV infection include recipient CMV-seropositivity, TCD of the graft, and acute GvHD (123). Early reconstitution of antiviral immunity remains an essential issue for the control of CMV reactivations after HSCT. The recovery of both CD8^+^ and CD4^+^ CMV-specific T cells may be a marker for protection against CMV reactivations (129).

Epstein–Barr virus infection is also a frequent viral complication after allogeneic HSCT, which may progress to EBV-associated posttransplant lymphoproliferative disease (PTLD) that causes unspecific symptoms such as fever and lymphadenopathy with a high viral load in the PB (130). These complications are mediated by several risk factors including TCD combined with reduced intensity conditioning (RIC) leading to impaired anti-EBV T cell-mediated immunity and persistence of residual recipient B cells (131). In addition, HLA disparity and acute GvHD have also been known to increase the risk of PTLD due to the delayed or impaired specific immune reconstitution (132).

However, other viral pathogens such as adenovirus (ADV), human herpes virus 6 (HHV6), BK-polyoma virus (BKV), and respiratory viruses occur less frequently in adult patients in comparison to CMV and EBV after allogeneic HSCT (133). The control of these viruses again depends upon the reconstitution of antiviral immunity.

### Antigen-Specific T Cell Reconstitution and Immunity Against CMV

**Figure 4:**
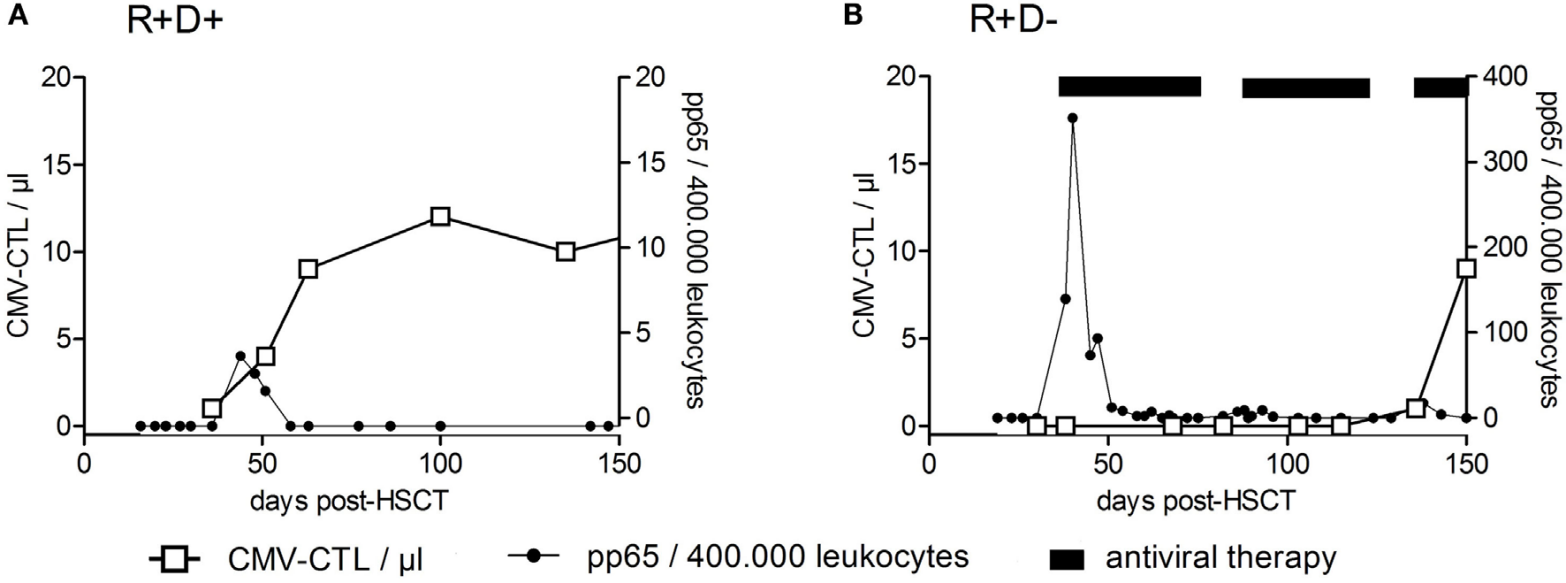
**Recovery of CMV-specific cytotoxic T lymphocytes after HSCT**. Examples of reconstitution of CMV-specific cytotoxic T lymphocytes (CTLs) after HSCT for CMV-seropositive recipients transplanted from CMV-seropositive donors (R+/D+) **(A)** and CMV-seropositive recipients transplanted from CMV-seronegative donors (R+D−) **(B)** are shown. CMV–CTL numbers per microliter of whole blood (left *y*-axis) were plotted against the time after HSCT (days). The right *y*-axis shows the number of pp65-positive cells/400,000 leukocytes (detection of CMV-reactivation). The CMV R+D+ patient had a CMV-reactivation by day +39 and responded by an expansion of CMV–CTLs. No significant reconstitution of CMV–CTLs within the CMV R+D− patient was detected until day +100 despite the early CMV reactivation. Adapted from Ref. (136).

Among the viruses mentioned above, T cell immune reconstitution against CMV has been studied most intensively and will be described in more detail below, as an example for virus (or any antigen)-specific T cell reconstitution and expansion. Apart from the above mentioned factors influencing T cell reconstitution (TCD of the graft; stem cell source, occurrence of acute or chronic GvHD), CMV serostatus of patient and donor is one of the most important variables influencing CMV-specific T cell immune reconstitution. CMV-seropositive recipients and donors (R+D+) have much faster reconstitution of CMV–CTLs (prior to day +50) and a subclinical CMV reactivation can even boost this development (106). On the other hand, CMV-seropositive recipients transplanted from CMV-seronegative donors (R+D−) lack the protective donor-derived immunity and hence have delayed recovery of antiviral immunity (between days +120 and +150) and a higher risk for recurrent CMV reactivations (134). In Figure 4, examples of patients with typical CMV–CTL immune reconstitution for R+D+ and R+D− groups are shown to demonstrate the impact of CMV serostatus on CMV–CTL immune reconstitution. Additionally, it has been shown, that CMV–CTLs of recipient origin can survive the conditioning regimen and can add to the protection against CMV, especially in R+D− patients (135).

#### Does the Quantity or Quality of CMV-Specific T Cells Matter?

Recent technological developments in cellular immunology have aided in the understanding of antigen-specific T cell responses and the antiviral immunity after HSCT. With the instigation of multimer (e.g., tetramer and streptamer) technology, antigen-specific T cells are readily detected and isolated without stimulation (137–139). In order to study those cell functions, we can choose from a broad variety of assays including cytokine secretion assays, ELISPOT, intracellular staining, which require stimulation of cells with viral lysates, viral proteins, or peptides (137). As for CMV, immunity toward CMV immunodominant epitopes, which include pp65 and IE-1 antigens have been most intensively studied (140, 141).

Initiation of multimer technology allowed the investigation of CMV–CTLs in patients after allogeneic HSCT in order to search for a protective threshold (113, 137, 142). A chronological overview of selected publications on monitoring of CMV-specific T cell responses after HSCT, with the focus on the protective numbers of CMV–CTLs is provided in Table 3. It has been shown that the inability to control CMV reactivation following HSCT is due to the impaired function of antigen-specific CD8^+^ T cells rather than an inability to recover sufficient numbers of CMV-specific T cells (143). Although CMV–CD8^+^ CTLs have been considered as the main antiviral effector cells, CMV-specific CD4^+^ T cells have been shown to play a crucial role in expansion and activation of CMV–CTLs, maintaining a long-term and efficient immunity against CMV (129). It has been reported that CD4^+^ and CD8+ CMV-specific T cells follow similar patterns of reconstitution (144), and their functional reconstitution is correlated with the absolute CD4^+^ or CD8^+^ T cell numbers (106, 145). So far, there is no threshold for protective levels of CMV-specific T cells applicable for all patients. Sequential monitoring of individual patients for the kinetics of CMV–CTL recovers, their ability to produce cytokines and expansion upon reactivation, are currently being used to detect recurrent CMV reactivations (136) (Figure 4). In summary, both the quantity and quality of immune reconstitution are important for preventing viral infection after allogeneic HSCT.

**Table 3:** **Selected publications on monitoring of CMV-specific T cell responses after HSCT**.

Reference	Key information
Altman et al. (146)	First use of MHC tetramers to enumerate and characterize antigen-specific T cells
Cwynarski et al. (113)	Protection from CMV reactivation with ≥10 CMV–CTL cells/μL blood
Gratama et al. (142)	(1) Failure to recover HLA-A*02-NLV–CMV–CTLs is associated with the development of CMV disease (2) Number of HLA-A*02-NLV–CMV–CTLs in the grafts administered to CMV-seropositive HSCT recipients is inversely correlated with the number of recurrent CMV infections
Aubert et al. (147)	Less than 20 cells/μL of HLA-A*02 CMV–CTLs predicted episodes of viral replication
Chen et al. (148)	More than 10–20 cells/μL CMV–CTLs conferred protection against CMV reactivation
Özdemir et al. (107)	Inability to control CMV reactivation is caused by impaired function of CMV–CTLs rather than an inability to recover sufficient numbers of CMV-specific T cells
Lacey et al. (149)	CMV-specific cellular immune responses restricted by HLA-B*07 dominated those restricted by HLA-A*02
Akiyama et al. (150)	Frequency of HLA-A*24 CMVpp65 tetramer-positive staining correlated with cytotoxicity and IFN-γ production
Bunde et al. (151)	High frequencies of IFN-γ producing IE-1, but not pp65-specific CD8^+^ T cells, correlated with protection from CMV disease
Lilleri et al. (152)	Levels of CD4^+^ T cells below 1 cell/μL and of CD8^+^ T cells less than 3 cells/μL did not protect against recurrent CMV infection
Gratama et al. (153)	(1) CMV–CTLs provided protection against recurrent CMV reactivations (2) CMV disease appeared to be prevented by the IE-1-specific subset rather than the pp65-specific CD8^+^ T cell subset
Koehl et al. (154)	(1) Numbers of CMV–CTLs differ significantly depending on the HLA type (2) Number of CMV–CTLs below 10 cells/μL does not correlate with susceptibility for CMV reactivation
Giest et al. (155)	HLA-A*24/pp65- and HLA-B*35/pp65-CTLs correlated with protection from CMV reactivation at significantly lower cell levels than HLA-A*01/pp50- and HLA-A*02/pp65-CTLs
Gratama, et al. (156)	Less than 7 cells/μL of CMV–CTLs during the first 65 days after transplantation was a significant risk factor for CMV-related complications
Borchers et al. (134)	(1) Presence of CMV–CTLs before day +50 and their expansion after reactivation protected against recurrent CMV reactivations (2) CMV–CTL reconstitution was delayed in the CMV R+D− group
Lilleri et al. (157)	Combination of CMV–CTL monitoring and viral monitoring can be used to direct preemptive treatment with antiviral drugs
Borchers et al. (136)	(1) 1 cell/μL of CMV–CTLs between days +50 and +75 marked the beginning of immune response against CMV in the CMV R+D+ group (2) Expansion of CMV (3) Sequential monitoring of CMV

*Reused from Ref. (137) by permission from Elsevier, License Number 3922460449459*.

*MHC, major histocompatibility complex; CMV–CTL, cytomegalovirus cytotoxic T lymphocytes; HLA, human leukocyte antigen; IFNγ, interferon gamma; IE-1, immediate early-1*.

## Conclusion and Future Outlook

Reconstitution of the donor-derived immune system is essential for control of infectious complications, modulation of GvHD, and relapse control, thus contributing to long-term survival. In this review, we have described the major events in immune cell reconstitution, considering the most important cell types, their approximate time of reconstitution, and their interaction after HSCT. The recovery of the innate immunity is vital, especially in the absence of CD4^+^CD45RO^+^ memory and CD4^+^CD45RA^+^ naive T cells.

Today, the understanding of CD4^+^CD25^+^CD127^−^ regulatory T cells has advanced significantly in both preclinical and clinical models for GvHD. The remaining challenge is to generate large amounts of CD4^+^CD25^+^CD127^−^ Tregs with high purity and stable Foxp3-expression in a cost effective way. A further clinical problem is the optimal time point of Treg application. If Tregs are administered to treat patients with steroid-refractory GvHD, there may be a substantial delay between production and application and, thus, lack of feasibility and treatment success. Furthermore, the impact of ongoing systemic immunosuppression on Treg cell function has to be considered in clinical trials. A further aspect to be solved in the future is to optimize tissue conditions for survival and expansion of T regs, as these cells are under the strong control of local microbiota especially in the main target tissues of GvHD.

## Author Contributions

EW, EH, and HG designed the review and revised it critically for important intellectual content. JO, MKJ, SG, and PV provided the draft, summarized available data, and selected the references. All the authors approved the final version of the manuscript.

## Conflict of Interest Statement

The authors declare that the research was conducted in the absence of any commercial or financial relationships that could be construed as a potential conflict of interest.
